# Quantum Oscillations at Integer and Fractional Landau Level Indices in Single-Crystalline ZrTe_5_

**DOI:** 10.1038/srep35357

**Published:** 2016-10-14

**Authors:** W. Yu, Y. Jiang, J. Yang, Z. L. Dun, H. D. Zhou, Z. Jiang, P. Lu, W. Pan

**Affiliations:** 1 Sandia National Laboratories, Albuquerque, New Mexico 87185, USA; 2 School of Physics, Georgia Institute of Technology, Atlanta, Georgia 30332, USA; 3 Department of Physics and Astronomy, University of Tennessee, Knoxville, Tennessee 37996, USA

## Abstract

A three-dimensional (3D) Dirac semimetal (DS) is an analogue of graphene, but with linear energy dispersion in all (three) momentum directions. 3D DSs have been a fertile playground in discovering novel quantum particles, for example Weyl fermions, in solid state systems. Many 3D DSs were theoretically predicted and experimentally confirmed. We report here the results in exfoliated ZrTe_5_ thin flakes from the studies of aberration-corrected scanning transmission electron microscopy and low temperature magneto-transport measurements. Several unique results were observed. First, a π Berry phase was obtained from the Landau fan diagram of the Shubnikov-de Haas oscillations in the longitudinal conductivity *σ*_xx_. Second, the longitudinal resistivity *ρ*_xx_ shows a linear magnetic field dependence in the quantum limit regime. Most surprisingly, quantum oscillations were also observed at fractional Landau level indices N = 5/3 and 7/5, demonstrating strong electron-electron interaction effects in ZrTe_5_.

Since the discovery of graphene^1^,^2^, Dirac materials have attracted tremendous attention due to their extraordinary electronic properties and great potential for applications in next generation electronic devices. Recently, research has extended to the search for three-dimensional (3D) Dirac semimetal (DS)^3^,^4^,^5^,^6^,^7^,^8^,^9^,^10^,^11^, where linear energy dispersion holds along all three momentum directions. Dirac semimetals have generated a great deal of current excitements and have made it possible to study quantum dynamics of relativistic field theory in a solid-state system^10^. Moreover, these topological materials are believed to be useful in future quantum information process.

Shortly after earlier theoretical predictions, many material systems have been experimentally confirmed to be 3D DSs. For example, using angle-resolved photoemission spectroscopy (ARPES), three groups were able to observe 3D Dirac fermions in Na_3_Bi and Cd_3_As_2_ single crystals^7^,^8^,^9^,^12^,^13^. Questions on whether ZrTe_5_ being a 3D DS, however, remain unsettled. In a recent theoretical study, it was shown that single-layer ZrTe_5_ is a quantum spin Hall insulator, while bulk ZrTe_5_ is very close to the phase transition boundary between a weak and strong topological insulator^14^. On the other hand, the electronic band structure measured by ARPES in ZrTe_5_ crystals is consistent with that expected for a 3D DS^10^. In addition to ARPES measurements, magneto-infrared spectroscopy at low temperatures in high magnetic fields further shows strong evidence of inter-Landau-level transitions resulting from Dirac fermions^11^,^15^. Quantum transport studies have also yielded intriguing results, such as the chiral magnetic effect^10^,^16^,^17^. To date, most of the transport studies in ZrTe_5_ were performed at relatively higher measurement temperatures, at which electron-phonon interactions can mask subtle correlation effects induced by strong electron-electron interactions. In this article, we show, by lowering the measurement temperature to 0.3 K, a striking observation of quantum oscillations at fractional Landau level (LL) indices.

## Results and Discussions

### ZrTe_5_ crystal structure by aberration-corrected scanning transmission electron microscopy

**Figure 1:**
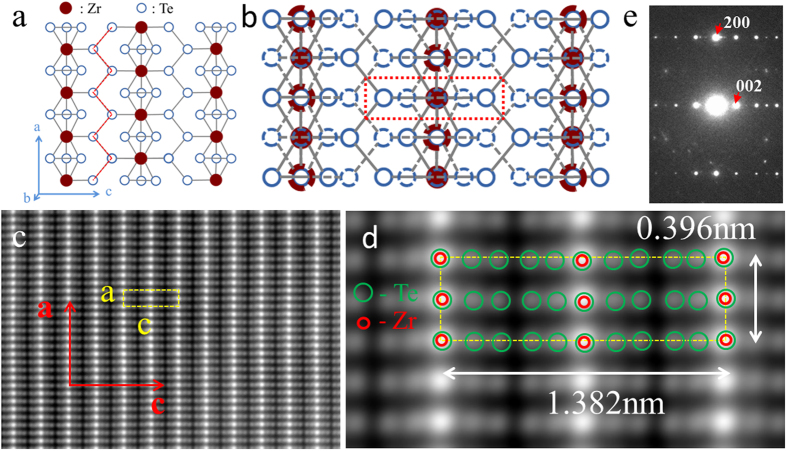
ZrTe_5_ crystal structure. (**a**) Schematic top view of a single-layer structure. The dashed red line shows the zigzag chain of Te (adapted from ref. 14). (**b**) Schematic top view of a two-layer structure. The solid patterns represent the top layer and the dashed the bottom layer. The bottom layer is shifted by a half lattice-constant along the [100]-crystal direction. Due to this shift, one Zr atom overlaps with a Te atom in the projection. (**c**) STEM high-angle annular dark-field (HAADF) image of ZrTe_5_ in [010] direction. (**d**) Zoomed-in image of the unit cell (dashed yellow rectangular) in (**c**). Red and green circles represent Zr and Te atoms, respectively. The bright spots are due to the overlapping of Zr and Te atoms in the projection. The measured lattice constant c = 1.382 nm and a = 0.396 nm. (**e**) Corresponding electron diffraction pattern in [010] direction with (200) and (002) planes as marked.

ZrTe_5_ has been studied in the past and is known for the resistivity anomaly and large thermoelectric power^18^,^19^. It is a layered material similar to graphite. The crystal structure^14^,^18^,^20^,^21^,^22^ contains chains of ZrTe_3_ prisms running parallel to the a-axis, as shown in Fig. 1a. These prismatic chains are linked along the c-axis via zigzag chains of Te atoms to form 2D planes, which stack along the b-axis. The layered structure of ZrTe_5_ distinguishes it from other 3D DSs. The weak van der Waals force between layers makes it easy to mechanically exfoliate thin flakes of ZrTe_5_. Figure 1c shows the projected arrangement of atoms in the a-c plane obtained from an aberration-corrected scanning transmission electron microscope (AC-STEM) taken with a high-angle annular dark-field (HAADF) detector. The high-resolution image in [010] projection in Fig. 1d reveals the atomic level details of the a–c plane of ZrTe_5_. To our knowledge, this is the first time a high-resolution STEM HAADF image has been taken for ZrTe_5_. The main difficulty of obtaining the STEM image is due to ZrTe_5_ being extremely sensitive to electron radiation. From the HAADF image, we were able to determine the lattice constants of the a–c plane, and a = 0.396 nm and c = 1.382 nm (Fig. 1d), which agree very well with the theoretical calculation^14^. The characteristic STEM pattern (i.e., an array of 5 spots with a bright one in the middle and four dim ones aside) can be explained by taking into account a half-lattice shift along the [100]-lattice direction between two adjacent layers in the [010] direction, as demonstrated in Fig. 1b. The electron diffraction pattern is shown in Fig. 1e. The crystal lattice constants deduced from this pattern are consistent with those obtained in Fig. 1d.

### Magneto-transport measurements

**Figure 2:**
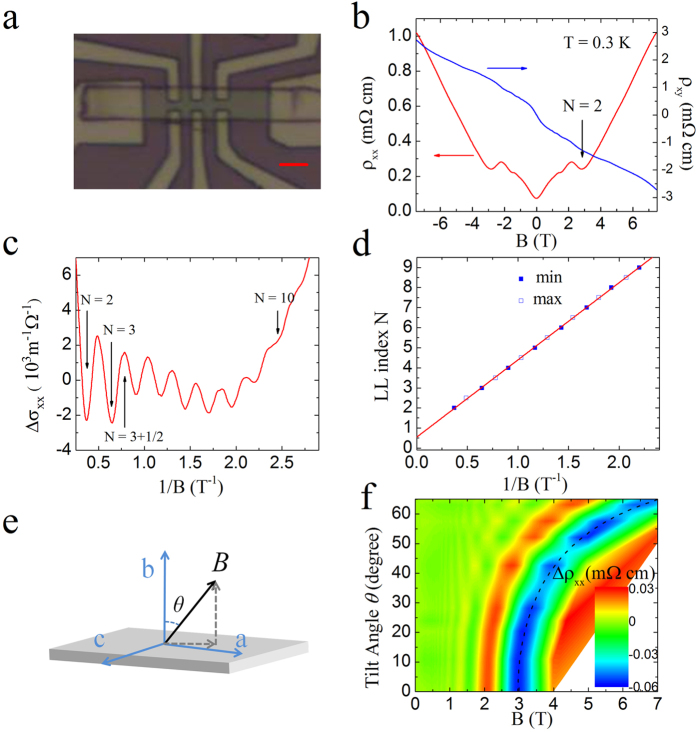
SdH oscillations in ZrTe_5_ and tilt magnetic field dependence. (**a**) Optical image of the Hall bar device. The scale bar is 5 μm. (**b**) The longitudinal resistivity (red line) and the transverse Hall resistivity (blue line) as a function of magnetic (*B*) field at *T* = 0.3 K. *B* is along b-axis, perpendicular to the cleavage plane. Well-developed SdH oscillations are observed. (**c**) *Δσ*_xx_ as a function of 1/*B* for tilt angle *θ* = 0°. Several LLs are labeled by the arrows. LL N = 10 corresponds to *B* ~ 0.4 T. (**d**) LL index N versus 1/*B*. The closed squares represent the integer index *Δσ*_xx_ minima and the open squares denote the half integer index *Δσ*_xx_ maxima. The solid line shows the best liner fit. An intercept of 0.55 is obtained, indicative of a π Berry phase. (**e**) Schematic of the sample in tilt magnetic field. (**f**) Contour plot of *Δρ*_xx_ with respect to tilt angle *θ* and magnetic field *B*. Blue color highlights the evolution of LL N = 2. The dashed line represents the best fit by 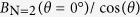, indicating the 2D-like transport behavior of charged carriers.

In Fig. 2a, we show a Hall bar device made of an exfoliated thin flake. The thickness of the flake is ~620 nm. Temperature (*T*) dependence of the longitudinal resistivity *ρ*_xx_ of our ZrTe_5_ thin flake at zero magnetic (*B*) field was measured and the details can be found in the Supplementary Figure S1. A characteristic resistivity peak is observed at T ~ 170 K, close to the values reported in previous works^17^,^23^. We note that the resistivity peak has recently been observed at a much lower temperature of ~60 K due to a lower impurity concentration^10^. Figure 2b shows the longitudinal magneto-resistivity *ρ*_xx_ and the Hall resistivity *ρ*_xy_ versus *B* (along b-axis, normal to the cleavage plane) at *T* = 0.3 K. Around *B* = 0, *ρ*_xx_ shows a positive, quadratic field dependence. As *B* continues to increase, strong quantum oscillations are observed. To analyze these quantum oscillations, we follow the standard practice^24^ and convert *ρ*_xx_ into *σ*_xx_ by using the formula *σ*_xx_ = *ρ*_xx_/(*ρ*_xx_^2^ + *ρ*_xy_^2^). In Fig. 2c, we display the oscillatory component (*Δσ*_xx_), after subtracting a smooth background from *σ*_xx_, as a function of 1/*B* at *T* = 0.3 K. The onset of the Shubnikov-de Haas (SdH) oscillations occurs at the Landau level index number N = 10 (corresponding to *B* ~ 0.4 T), though weak undulations are still visible up to N = 12 (or *B* ~ 0.34 T). From the criterion of 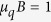 for the onset of SdH oscillations, a quantum mobility of 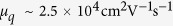 is obtained. A similar value was also reported recently^17^. Beyond the SdH oscillation at *B* ~ 2.7 T (corresponding to the N = 2 Landau level index), *ρ*_xx_ assumes a linear-in-*B* field dependence. We note here that a linear magneto-resistance (MR) has been observed for massless Dirac fermions in the quantum limit^25^,^26^,^27^,^28^, due to their linear energy dispersion. We believe this mechanism is also responsible for the linear-in-*B* dependence in our sample. The Hall resistivity shows a negative slope, from which we conclude that the carriers are electrons in our device. In the high *B* field regime, the Hall resistivity is generally linear with undulations concomitant with the SdH oscillations. It deviates slightly from the linear field dependence around ~3 T, as the quantum limit is approached. The linear fit to *ρ*_xy_ in the *B* field range of 1 T < *B *< 3 T yields a Hall density of 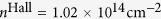 (more details can be found in Supplementary Figure S2). We note that this deduced *n*^Hall^ is much higher than the electron density 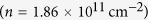 obtained from the SdH oscillations analysis (as we show below), indicating the co-existence of a two-dimensional (2D) electron system on the surface of ZrTe_5_ and three-dimensional bulk carriers. This two-carrier model is also consistent with the positive MR around *B* = 0 T^29^. Furthermore, the large bulk density probably is responsible for the non-zero value of the *R*_xx_ minima in the SdH oscillations, either due to an incomplete bulk localization or due to the coupling between the bulk and the 2D conducting layer^30^. The Hall slope is larger around zero magnetic field. Similar behavior was also reported in the past, and attributed to either a two-carrier transport^31^ or a topological effect^32^.

To understand whether the SdH oscillations are of 2D nature, we carried out magneto-transport measurements in tilt magnetic fields. The tilt angle *θ* between *B* and b-axis can be varied from 0 to 90° as schematically shown in Fig. 2e. The angle is calibrated by measuring the quantum oscillations of an InAs quantum well that was mounted together with ZrTe_5_ on the same chip carrier. In order to better reveal the SdH oscillations as a function of title angle, we subtract a smooth background from the measured longitudinal resistivity *ρ*_xx_. Figure 2f shows the contour plot of *Δρ*_xx_ as a function of *B* at various tilt angle *θ*. The oscillation extrema shift to higher magnetic field systematically with increasing *θ*. Indeed, the magnetic field position of the N = 2 Landau level versus *θ* can be well fitted using 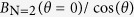 as shown by the dashed line in Fig. 2f, indicating that the electrons that contribute to the SdH oscillations are of 2D nature.

Next, we follow the well-developed methodology and construct the Landau fan diagram. To do this, we assign a Landau level index number N (N + 1/2) to each *Δσ*_xx_ minimum (maximum)^33^, as shown in Fig. 2c. In the whole field region, N increases by 1 between the adjacent oscillations, indicating the spin degeneracy is not lifted in our device. In the Landau fan diagram (Fig. 2d), the data points fall on a straight line and the solid line represents the best linear fit. The linear extrapolation gives an intercept of 0.55^24^, implying a non-zero Berry phase and the Dirac nature of the electrons responsible for the SdH oscillations. The same conclusion has also been reached in other studies, such as magneto-infrared^15^, ARPES^10^, and magneto-transport^11^. From the slope of the linear fit in Fig. 2d, a 2D carrier density of 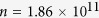 cm^−2^ is deduced.

**Figure 3:**
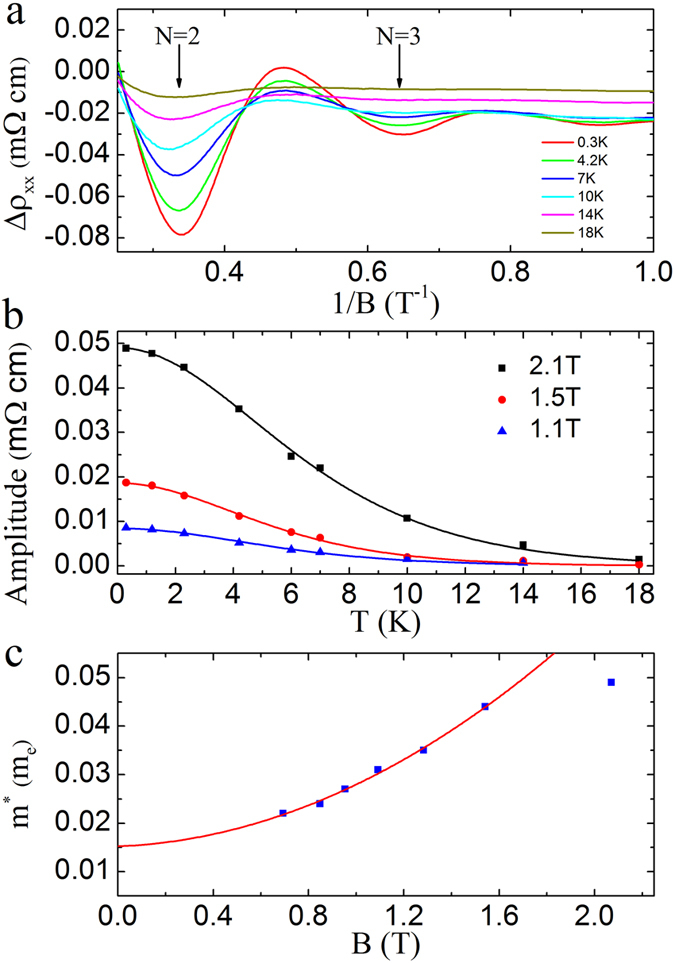
Determining effective mass from temperature dependence of SdH oscillations. (**a**) *Δρ*_xx_ as a function of 1/*B* at different temperatures obtained by subtracting a smooth background. LLs N = 2, 3 are labeled by the arrows. (**b**) SdH oscillation amplitude as a function of temperature at different magnetic fields. The solid lines represent the best fits using equation (1). Data points obtained at low fields can be found in the Supplementary Figure S5. (**c**) Extracted effective mass at different magnetic fields. The line is a quadratic magnetic field dependent fit.

We now deduce the effective mass m^*^ of the 2D carriers from the temperature dependence of the well-developed SdH oscillations (Fig. 3a). The SdH oscillation amplitude can be described by the Lifshitz-Kosevich equation^1^


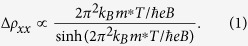


In equation (1), *k*_*B*_ is the Boltzmann’s constant, 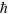 is the reduced Plank’s constant, and *e* is the electron charge. Figure 3b shows the oscillation amplitudes as a function of temperature for different magnetic fields. The effective mass can be extracted by fitting the temperature dependence of the oscillation amplitude (solid lines in Fig. 3b). In Fig. 3c, m^*^ is plotted as a function of magnetic field. It shows strong magnetic field dependence. In fact, it follows generally a quadratic *B* dependence, and increases from 0.022 *m*_*e*_ (*m*_*e*_ is the electron rest mass) at *B* ~ 0.7 T to 0.044 *m*_*e*_ at *B* ~ 1.5 T. This strong *B* dependence of m^*^ is unexpected, and its origin is not known to us at the present time. On the other hand, it is known that the Lifshitz-Kosevich equation works better in the low magnetic fields limit. Following the quadratic *B* dependence, the zero *B* field effective mass, m^*^ = 0.015 *m*_*e*_, can be deduced. The effective mass at *B* = 2.1 T apparently deviates from this quadratic dependence, and is smaller. One possible reason for a smaller mass at this *B* field is due to a relatively large Zeeman splitting. Indeed, the effective g-factor in ZrTe_5_ was deduced to be around ~16–22^15^. At *B* = 2.1 T, the Zeeman splitting is about 23–31 K, comparable to the Landau level disorder broadening (*Γ* ) of 30 K. Here the Landau level broadening is estimated from the quantum mobility and 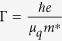 ~ 30 K. The contribution from two partially spin resolved Landau levels may be responsible for a smaller effective mass at *B* = 2.1 T.

With the zero field m^*^ and 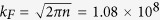 m^−1^, the Fermi velocity 
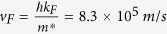
 and the Fermi level 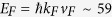 meV are deduced for our sample. We note here that the obtained *v*_*F*_ is higher than those reported in refs 11 and 15 from magneto-infrared measurements, but comparable to that in ref. 10 obtained in the ARPES measurements.

**Figure 4:**
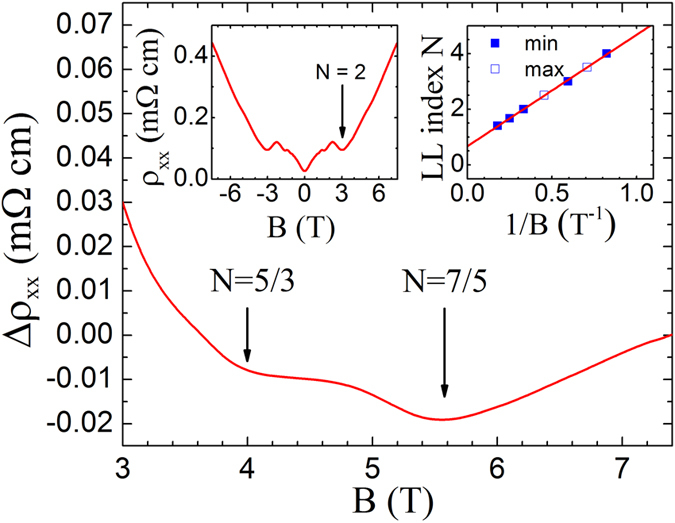
Quantum oscillations at fractional Landau level indices. *Δρ*_xx_ of 1.35-μm-thick sample in the quantum limit regime is plotted as a function of magnetic field at *T* = 0.3 K. Here, *Δρ*_xx_ is obtained by subtracting a linear background. Fractional Landau level indices at N = 5/3 and 7/5 are labeled by the arrows. Inset: left inset shows the *ρ*_xx_ data. Right inset: Landau fan diagram obtained from 1.35-μm-thick sample. The intercept of 0.67 is extracted from the best linear fit.

In the following, we present the most striking result in our experiment – the observation of quantum oscillations at fractional Landau level indices in the quantum limit regime. In the left inset of Fig. 4, we plot *ρ*_xx_ of a second sample (thickness ~1.35 μm). Quantum oscillations, similar to those in the thinner sample, are observed. In Fig. 4, we plot *Δρ*_xx_ (after subtracting a linear background) in the high magnetic field regime at *T* = 0.3 K. Additional minima at *B* ~ 4 T, and 5.6 T are clearly visible beyond the N = 2 quantum oscillation. These minima are considerably weaker than those at integer Landau level indices. We point out here that these minima are not experimental artifacts, as they were reproducible in different cool-downs of the same sample, and were also observed in the thinner sample (more details can be found in Supplementary Figure S6). The fractional Landau level indices of N = 5/3 and 7/5 can be assigned to these two minima, respectively, as determined from the Landau fan diagram (the right inset of Fig. 4). Similar anomalous resistivity minima at fractional Landau level indices were also observed in the quantum limit regime in a topological insulator of Bi_2_Se_3_^34^. There, they were viewed as the precursors to the fractional quantum Hall effect (FQHE). Following the same argument, we shall also attribute these resistivity minima with fractional Landau level indices in our ZrTe_5_ sample as the developing FQHE states. The occurrence of these developing FQHE states represents compelling evidence of the electron-electron interactions induced many-body effects in ZrTe_5_. The right inset of Fig. 4 shows the Landau fan diagram, including the two data points at N = 5/3 and 7/5. An intercept of 0.67 is obtained at 1/*B* = 0. This non-zero intercept is consistent with that in the thinner sample and, again, implies a non-zero Berry phase. We note here that the value of the intercept in this thicker sample is larger than that in the thinner sample. This might be due to the additional phase shift of ±1/8 in this thicker, 3D-like sample^35^.

In summary, for the first time, high resolution HAARD images in ZrTe_5_ were obtained. Moreover, Shubnikov-de Haas oscillations were observed in high quality ZrTe_5_ thin flakes and a non-zero Berry phase was obtained in the Landau fan diagram, manifesting the Dirac fermion nature of the charged carriers in ZrTe_5_. Most surprisingly, quantum oscillations were observed at the fractional Landau level index numbers N = 5/3 and 7/5 in the quantum limit regime, manifesting novel quantum phenomena induced by strong electron-electron interactions in ZrTe_5_.

## Methods

### ZrTe_5_ synthesis

ZrTe_5_ polycrystalline sample was prepared by reacting appropriate ratio of Zr and Te in a vacuumed quartz tube at 450 degree for one week. The ZrTe_5_ single crystal sample was prepared by chemical vapor transport technique^36^,^37^. The transport agent is iodine and the transport temperature is from 530 degree to 450 degree. The transport time is around 20 days.

### Device fabrication

By mechanically exfoliating ZrTe_5_ single crystal, thin flakes were transferred onto 1-μm-thick silicon dioxide on P-Si <100> substrate. 300-nm-thick Pd electrodes were defined using e-beam lithography technique followed by physical vapor deposition. A standard lift-off process is employed.

### Characterization

The thickness of thin ZrTe_5_ flake was measured using atomic force microscope (Veeco D3100 with a Nanoscope IVA Controller).

### Transport measurement

The measurement was performed in a He-3 cryostat equipped with a superconducting magnet. The magnetic field can go up to 7.5 T. A standard low-frequency lock-in technique is used to measure the resistance with an *ac* excitation current of 5 μA. Angular-dependent measurement was carried out by mounting the sample on a rotary stage. ZrTe_5_ and InAs quantum well samples were put side by side on the same chip carrier. The tilt angle was calibrated by measuring the quantum oscillations of InAs.

## Additional Information

**How to cite this article**: Yu, W. *et al*. Quantum Oscillations at Integer and Fractional Landau Level Indices in Single-Crystalline ZrTe_5_. *Sci. Rep.* **6**, 35357; doi: 10.1038/srep35357 (2016).

## Supplementary Material

Supplementary Information
