# Supplementary material for: Quantum Oscillations at Integer and Fractional Landau Level Indices in Single-Crystalline ZrTe5
Source: Sci Rep. 2016 Oct 14;6:35357. doi: 10.1038/srep35357 (PMC5064405; doi:10.1038/srep35357)
Supplement: Supplementary Information [file srep35357-s1.pdf]

Supplementary Information

Quantum Oscillations at Integer and Fractional Landau Level Indices in Single-Crystalline $ZrTe_5$

W. Yu[1,*], Y. Jiang[2], J. Yang[2], Z. L. Dun[3], H. D. Zhou[3], Z. Jiang[2], P. Lu[1], and W. Pan[1]

[1]*Sandia National Laboratories, Albuquerque, New Mexico 87185, USA*

[2]*School of Physics, Georgia Institute of Technology, Atlanta, Georgia 30332, USA*

[3]*Department of Physics and Astronomy, University of Tennessee, Knoxville, Tennessee 37996, USA*

*Corresponding author: wnyu@sandia.gov

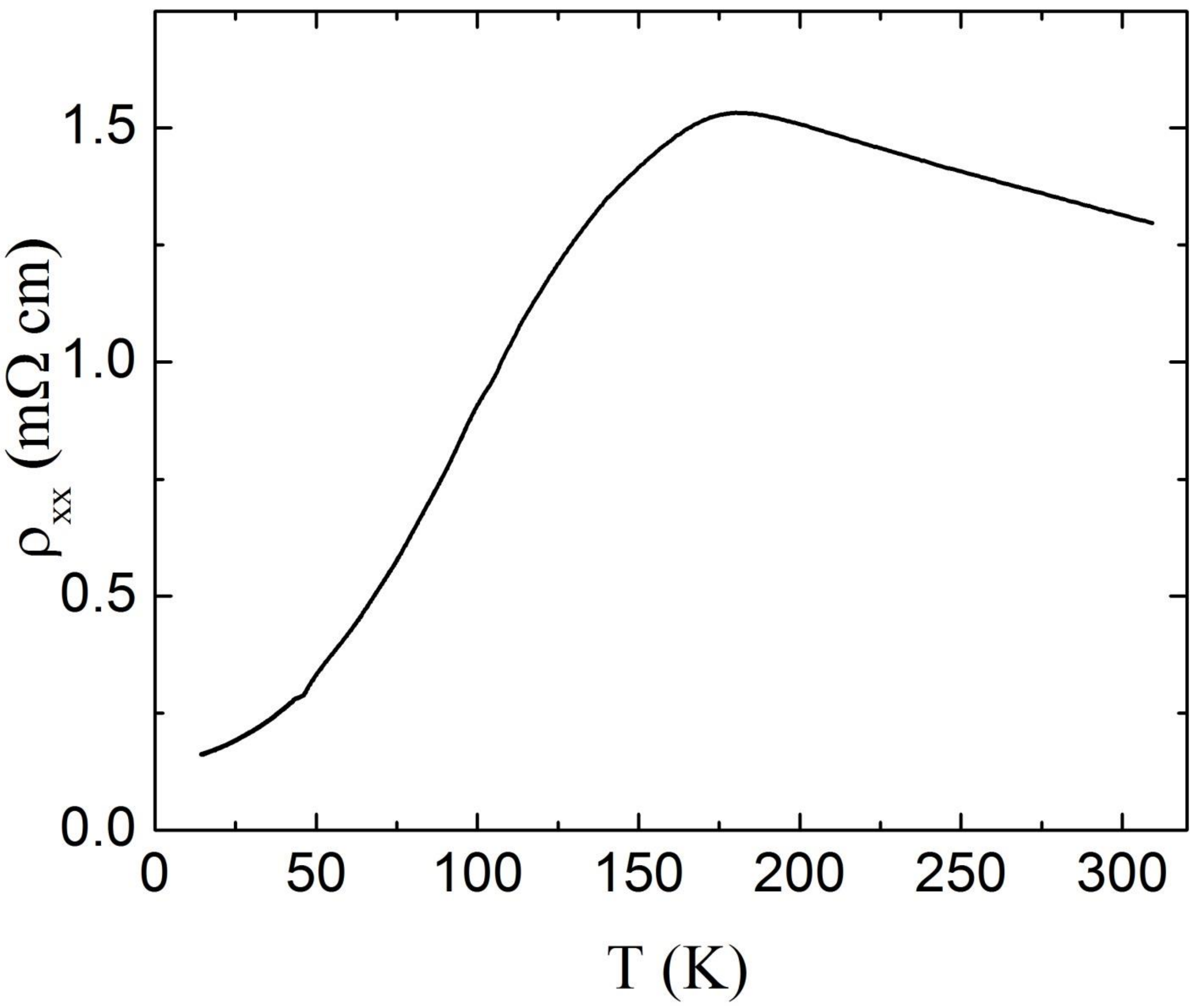

Figure S1. The temperature dependence of the longitudinal resistivity ρ_{xx} of $ZrTe_5$ thin flake at zero magnetic field. A characteristic resistivity peak is observed at T~170 K, close to the values reported in previous works[1,2].

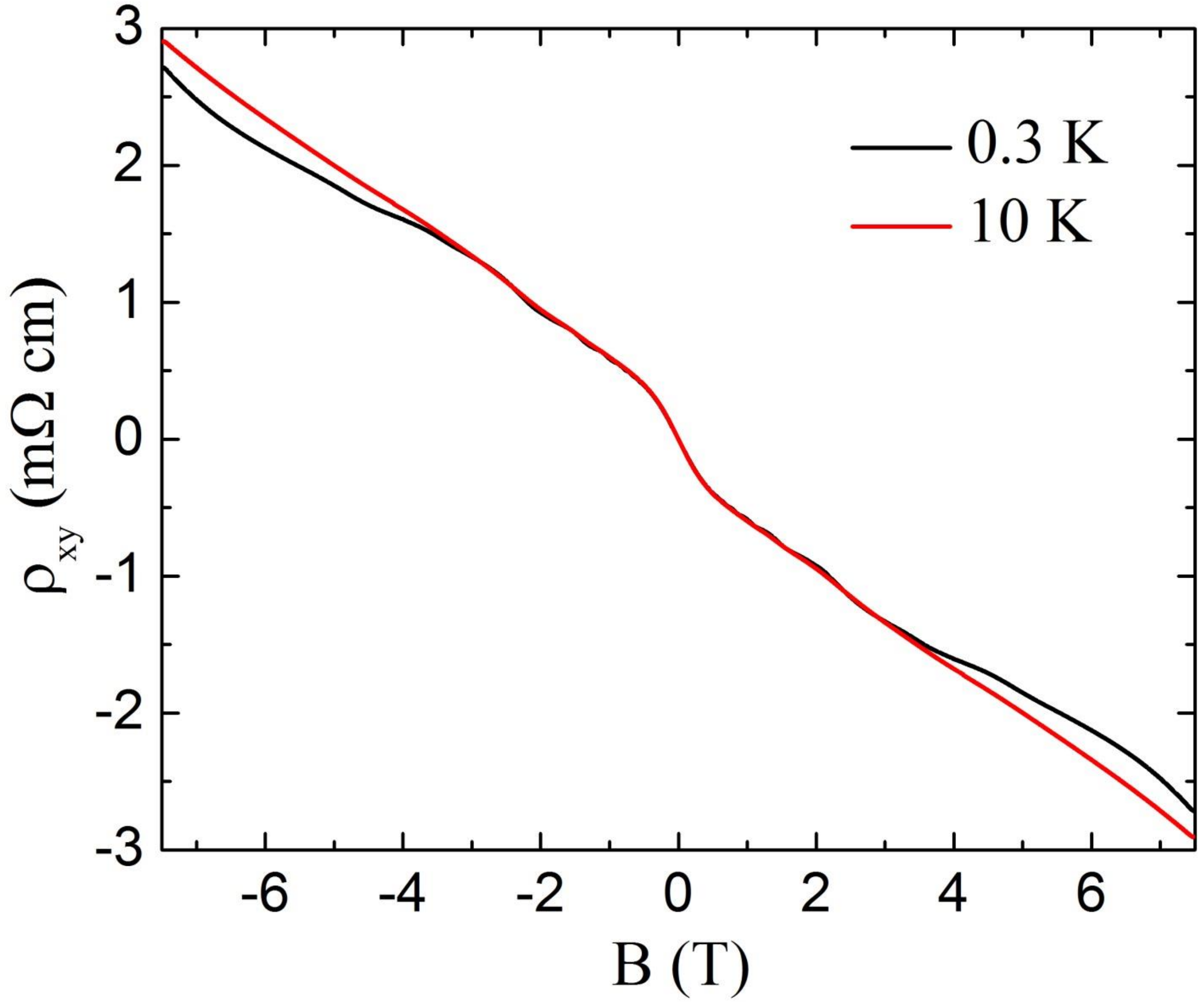

Figure S2. Hall resistivity at T = 0.3 K and T = 10 K, respectively. The overlap of these two traces between -3T and 3T indicates a constant carrier density in this large temperature range. Due to the deviation of ρ_{xy} at 0.3 K above 3T and the Hall anomaly below 1 T, we perform a linear fit to ρ_{xy} in the range of 1T < B< 3T to extract a more reliable carrier dentisy.

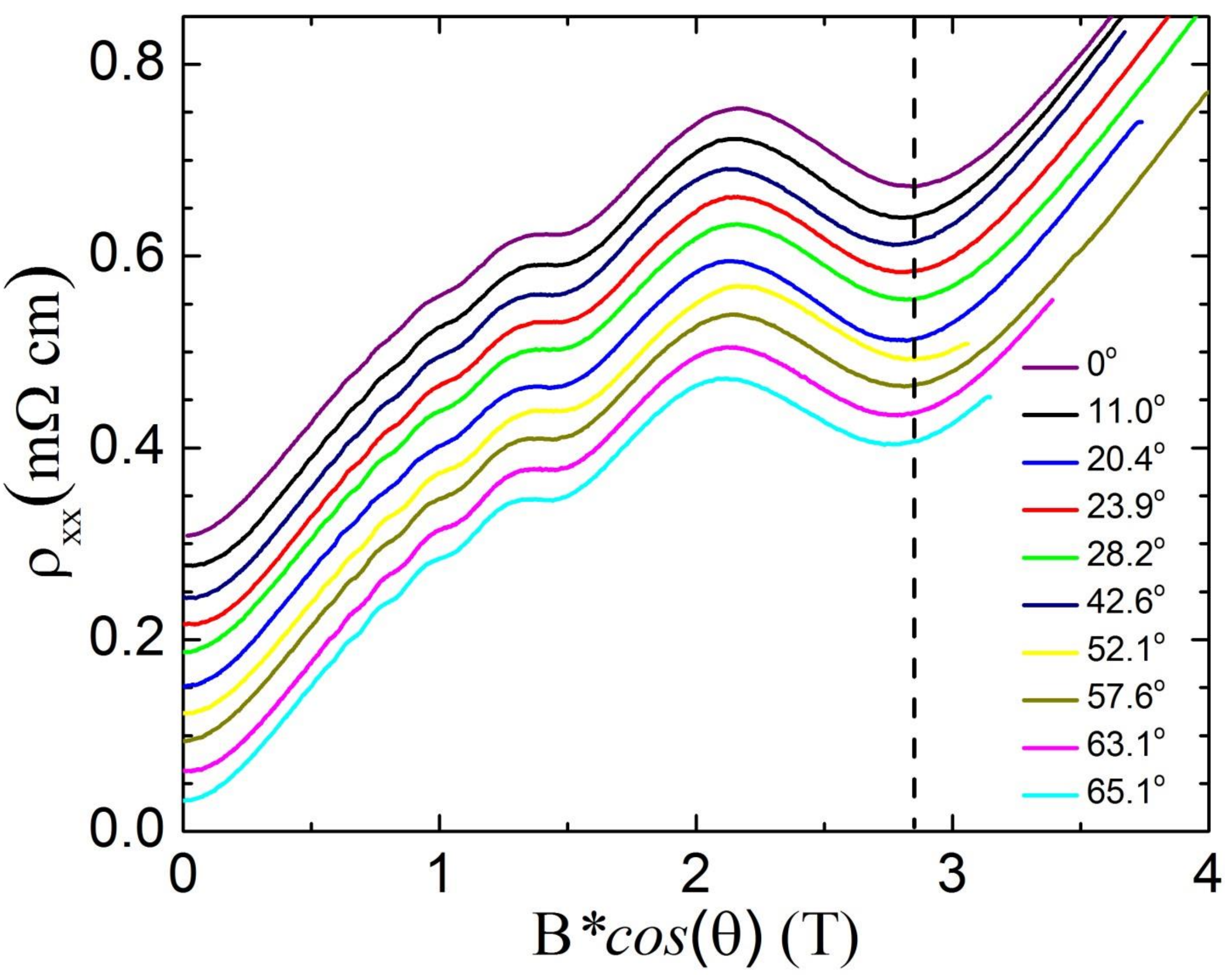

Figure S3. Angular-dependent measurement at T = 0.3 K. ρ_{xx} is plotted against $B_{\perp} = Bcos(\theta)$. The dashed line indicates the N = 2 Landau level. Curves are shifted vertically for clarity. From this data, we can conclude that the electrons that contribute to the SdH oscillations are of 2D nature.

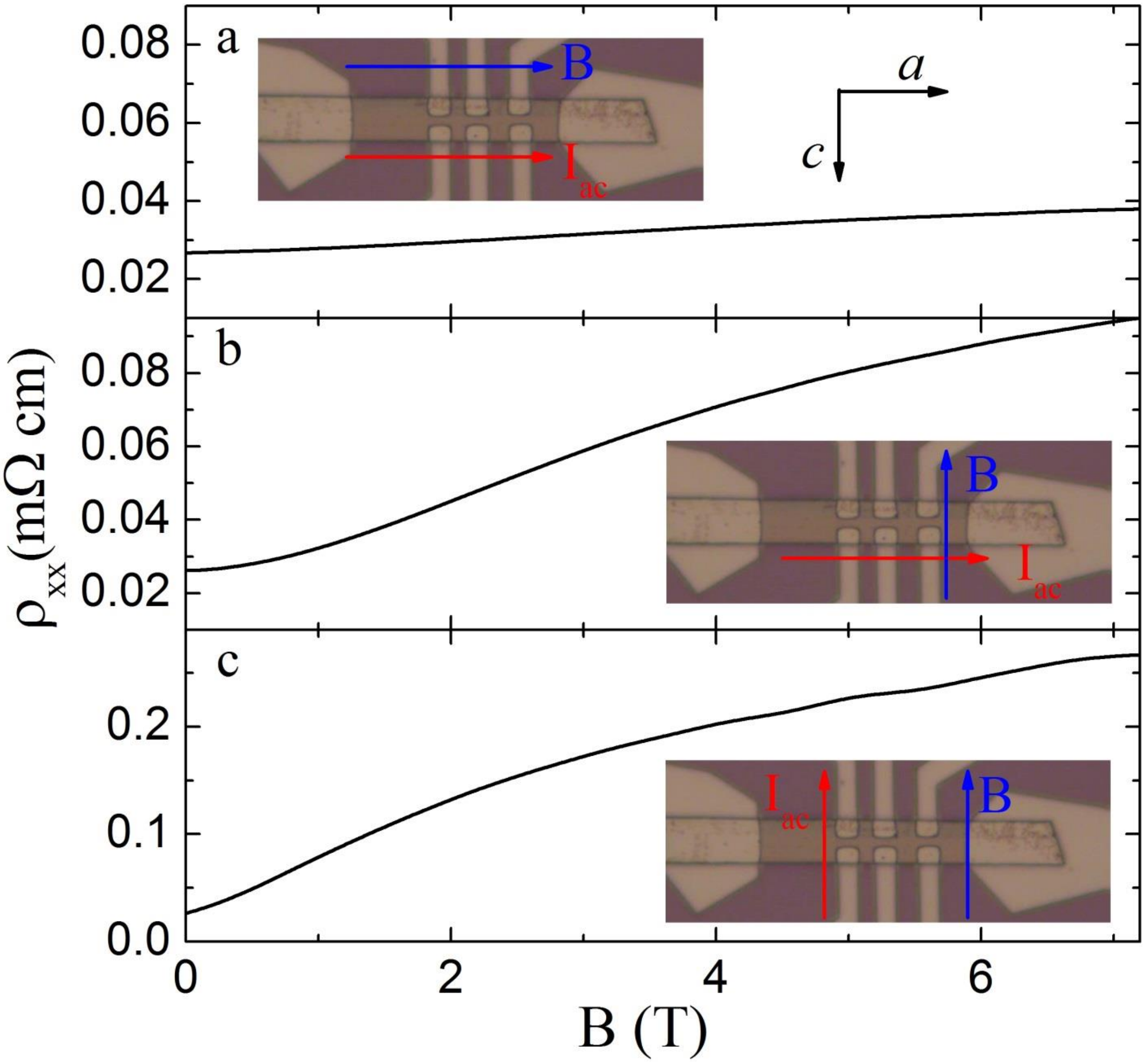

Figure S4. Magnetoresistance of the 1.35-μm-thick sample with in-plane magnetic field (*B* // ac plane). (a) ρ_{xx} versus *B* curve. *B* is parallel to the ac current I_{ac} along a-axis direction. (b) ρ_{xx} versus *B* curve. *B* is parallel to c-axis while I_{ac} is in the a-axis direction. (c) ρ_{xx} versus *B* trace in the case that *B* and I_{ac} are along c-axis.

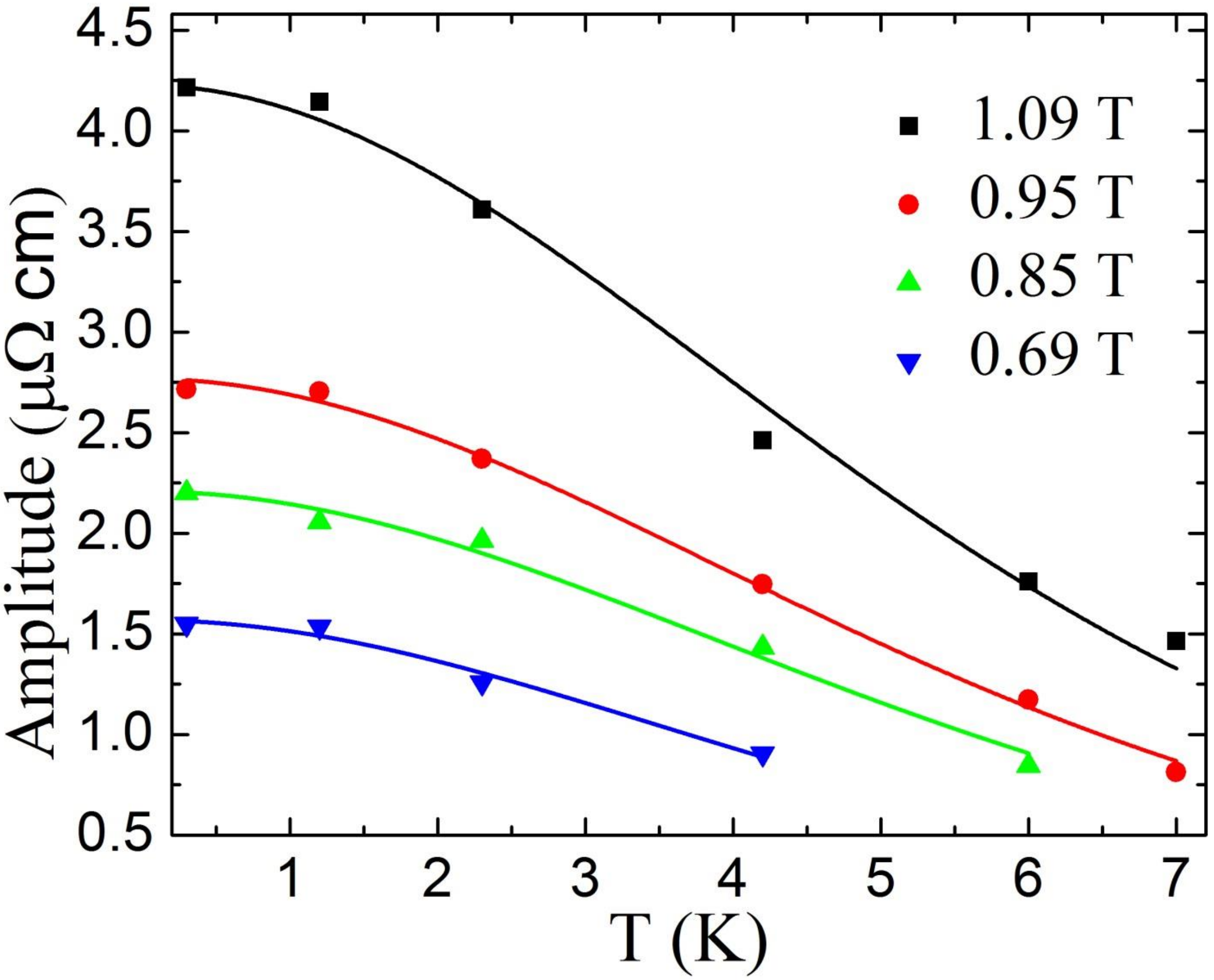

Figure S5. SdH oscillation amplitude as a function of temperature in the low magnetic field regime. The solid lines represent the best fits using equation (1).

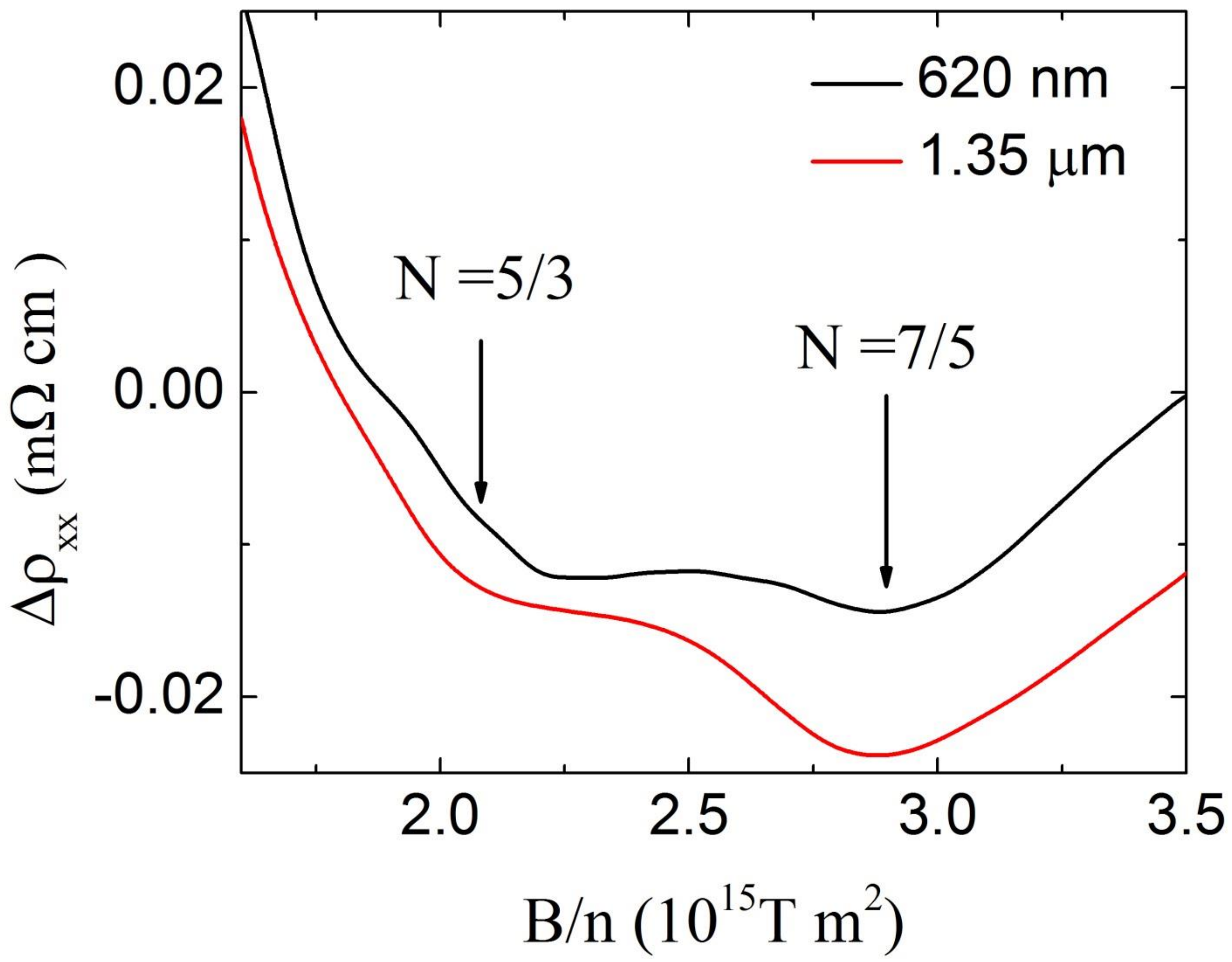

Figure S6. Quantum oscillations at fractional Landau level indices. $\Delta\rho_{xx}$ in the quantum limit regime is plotted as a function of normalized magnetic field B/n for two samples, where n is the carrier density extracted from the Shubnikov-de Haas oscillations. $\Delta\rho_{xx}$ is obtained by subtracting a linear background. Fractional Landau levels at N = 5/3 and N = 7/5 are marked by arrows. The red line shows $\Delta\rho_{xx}$ of the 1.35-μm-thick sample, while the black line represents the thinner sample of 620 nm thickness. The minimum at N = 5/3 for the thinner sample is relatively weak and broad.

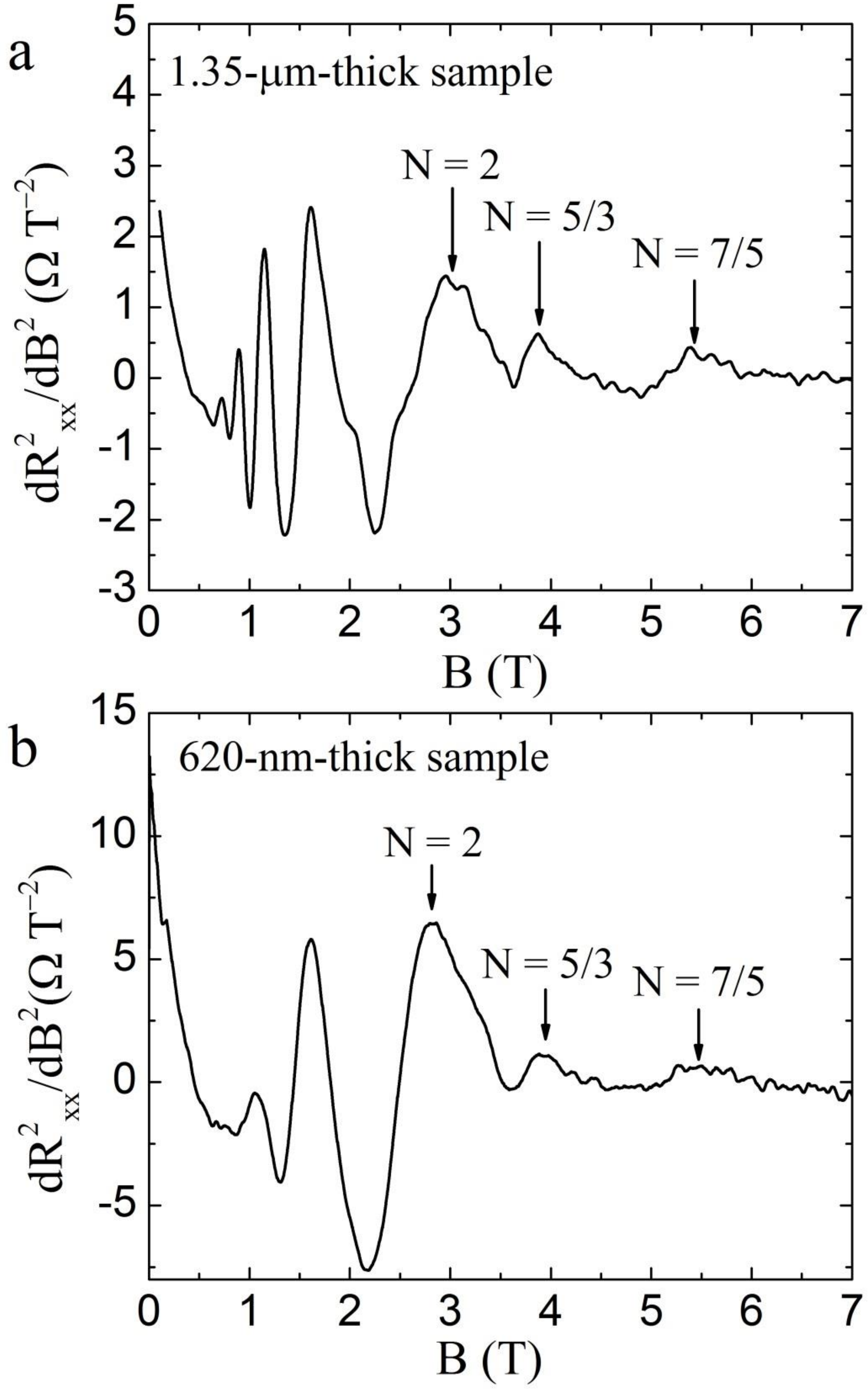

Figure S7. dR^2_{xx}/dB^2 as a function of magnetic field B shows quantum states at integer and fractional Landau level indices for both samples.

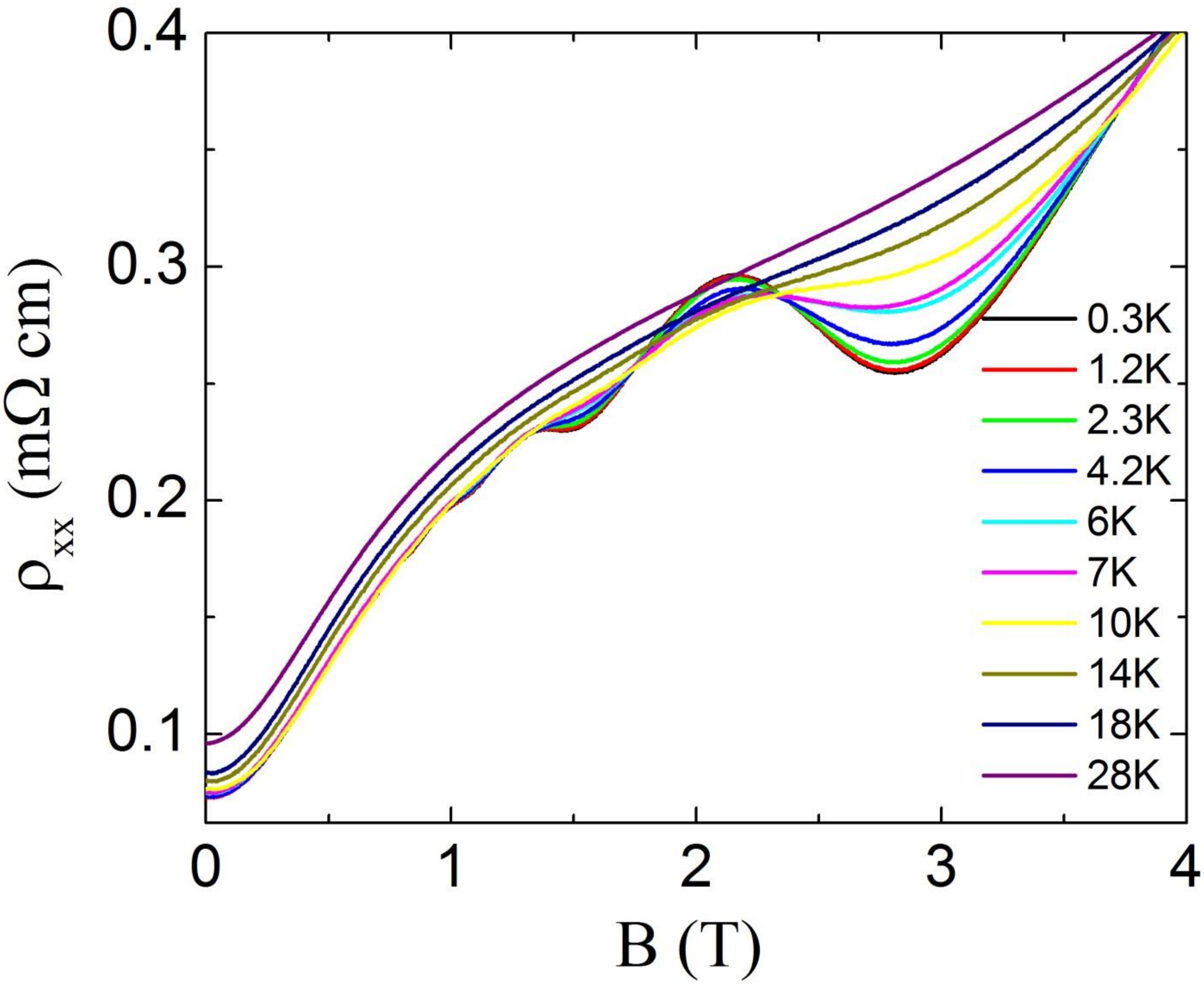

Figure S8. ρ_{xx} versus *B* at different temperatures in a perpendicular magnetic field. Quantum oscillations disappear at 28K. The ρ_{xx} trace at 28K is used as the background to study the oscillations at low temperature.

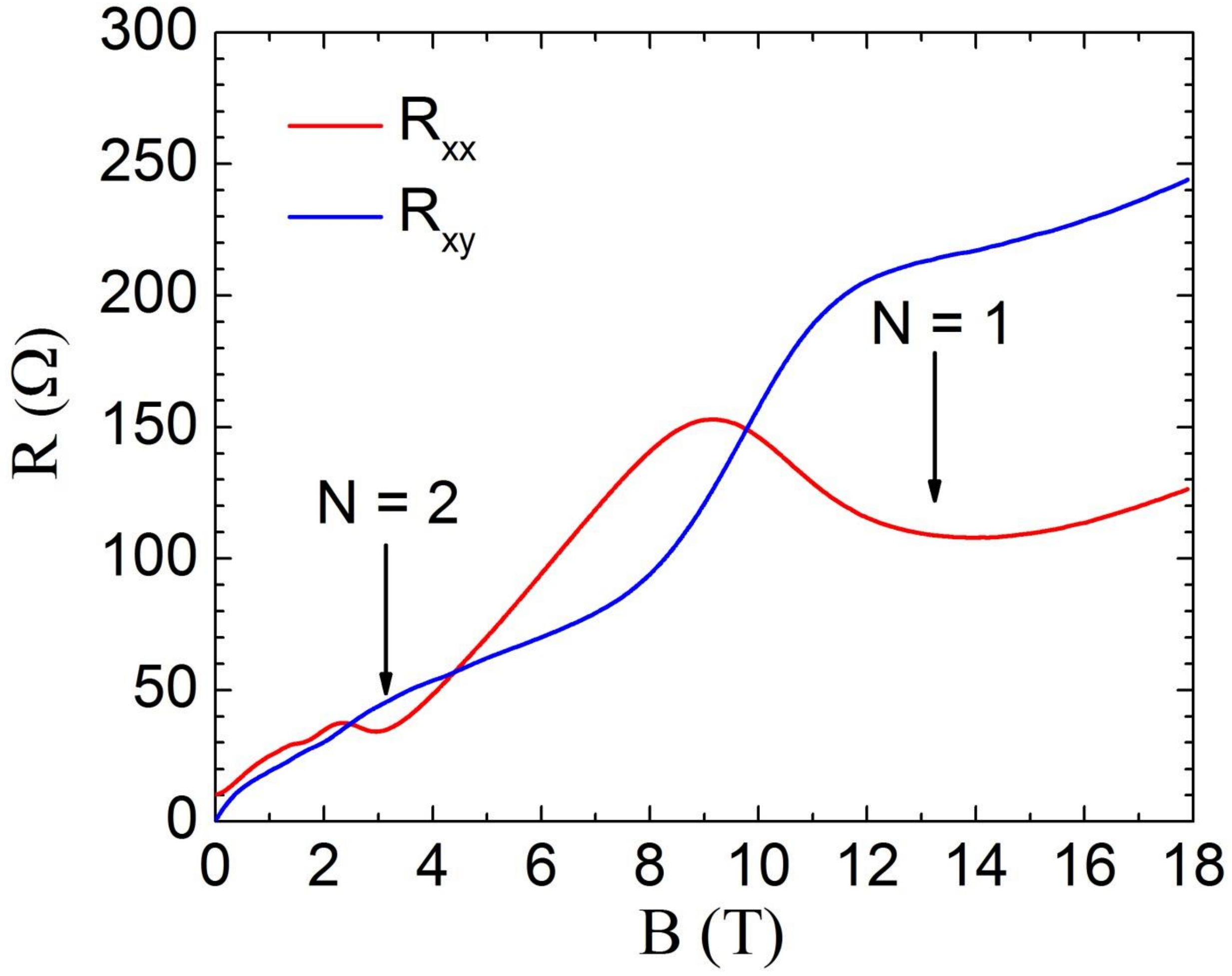

Figure S9. Measurement result of a third sample at 20mK. R_{xx} shows linear-in-*B* dependence in the range 3T < *B* < 11T. Another strong quantum state appears at ~ 13T which corresponds to Landau level N = 1.

References

1 Okada, S., Sambongi, T., Ido, M. Giant resistivity anomaly in $ZrTe_5$. *J. Phys. Soc. Jpn.* **49**, 839-840, doi:10.1143/JPSJ.49.839 (1980).

2 Stillwell, E. P., Ehrlich, A. C., Kamm, G. N., Gillespie, D. J. Effect of elastic tension on the electrical resistance of $HfTe_5$ and $ZrTe_5$. *Phys. Rev. B* **39**, 1626-1632 (1989).
